# Efficacy of *beet*root juice on reducing blood pressure in hypertensive adults with autosomal dominant *p*olycystic *k*idney *d*isease (BEET-PKD): study protocol for a double-blind, randomised, placebo-controlled trial

**DOI:** 10.1186/s13063-023-07519-2

**Published:** 2023-07-29

**Authors:** Priyanka S. Sagar, Alexandra Munt, Sayanthooran Saravanabavan, Farnoosh Asghar Vahedi, James Elhindi, Beatrice Nguyen, Katrina Chau, David C. Harris, Vincent Lee, Kamal Sud, Nikki Wong, Gopala K. Rangan

**Affiliations:** 1 Michael Stern Laboratory for Polycystic Kidney Disease, Westmead Institute for Medical Research, The University of Sydney, Sydney, NSW 2145 Australia; 2 Department of Renal Medicine, Westmead Hospital, Western Sydney Local Health District, Sydney, NSW 2145 Australia; 3 Research and Education Network, Westmead Hospital, Western Sydney Local Health District, Sydney, NSW 2145 Australia; 4 Department of Renal Medicine, Blacktown Hospital, Western Sydney Local Health District, Sydney, NSW 2148 Australia; 5 Blacktown Clinical School, Western Sydney University, Blacktown, NSW 2148 Australia; 6 Faculty of Medicine and Health, The University of Sydney, Sydney, NSW 2145 Australia; 7 Department of Renal Medicine, Nepean Hospital, Nepean Blue Mountains Local Health District, Sydney, NSW 2750 Australia

**Keywords:** Polycystic kidney disease, Beetroot juice, Dietary interventions in hypertension

## Abstract

**Background:**

In autosomal dominant polycystic kidney disease (ADPKD) impaired nitric oxide (NO) synthesis, in part, contributes to early-onset hypertension. Beetroot juice (BRJ) reduces blood pressure (BP) by increasing NO-mediated vasodilation. The aim of this double-blind, randomised, placebo-controlled study is to test the hypothesis that BRJ reduces systolic and diastolic clinic BP in hypertensive adults with ADPKD.

**Methods:**

Participants with ADPKD and treated hypertension (*n* = 60) will be randomly allocated (1:1) to receive a daily dose of either nitrate-replete (400 mg nitrate/day) or nitrate-deplete BRJ for 4 weeks. The co-primary outcomes are change in mean systolic and diastolic clinic BP before and after 4 weeks of treatment with daily BRJ. Secondary outcomes are changes in daily home BP, urinary albumin to creatinine ratio, serum and salivary nitrate/nitrite levels and serum asymmetric dimethylarginine levels before and after 4 weeks of BRJ.

**Discussion:**

The effect of BRJ in ADPKD has not been previously tested. BRJ is an accessible, natural dietary supplement that, if effective, will provide a novel adjunctive approach for treating hypertension in ADPKD.

**Trial registration:**

ClinicalTrials.gov NCT05401409. Retrospectively registered on 27th May 2022.

**Supplementary Information:**

The online version contains supplementary material available at 10.1186/s13063-023-07519-2.

## Administrative information

This table and the following study protocol contain all administrative information as required by the SPIRIT reporting guidelines and World Health Organization Trial Registration Data Set for clinical trial registration [1].

**Table:** 

Data category	Information
Public and Scientific Title	Double-blind, randomised, placebo-controlled study to determine the effect of beetroot juice on reducing blood pressure in hypertensive adults with autosomal dominant polycystic kidney disease
Primary registry and trial identifying number	NCT05401409 [ClinicalTrials.gov]. Registered on 27th May 2022 There are no secondary identifying numbers
Protocol Version	Version 7 (approved on 27^th^ June 2022)
Funding	This study was funded by a grant from PKD Australia to GR. PS was supported by an ICPMR Jerry Koutts Scholarship. The funding bodies have no role in the study design, execution, analyses, interpretation of the data, or decision to submit results
Author details	1. Priyanka S Sagar, Michael Stern Laboratory for Polycystic Kidney Disease, Westmead Institute for Medical Research (WIMR), The University of Sydney (USyd), Australia & Department of Renal Medicine, Westmead Hospital, Western Sydney Local Health District (WSLHD), Australia 2. Alexandra Munt, Michael Stern Laboratory for Polycystic Kidney Disease, WIMR, USyd, Australia & Department of Renal Medicine, Westmead Hospital, WSLHD, Australia 3. Sayanthooran Saravanabavan, Michael Stern Laboratory for Polycystic Kidney Disease, WIMR, USyd, Australia & Department of Renal Medicine, Westmead Hospital, WSLHD, Australia 4. Farnoosh Asghar Vahedi, Michael Stern Laboratory for Polycystic Kidney Disease, WIMR, USyd, Australia & Department of Renal Medicine, Westmead Hospital, WSLHD, Australia 5. James Elhindi, Research and Education Network, Westmead Hospital, WSLHD, Australia 6. Beatrice Nguyen, Michael Stern Laboratory for Polycystic Kidney Disease, WIMR, USyd, Australia 7. Katrina Chau, Department of Renal Medicine, Blacktown Hospital, WSLHD, Australia & Blacktown Clinical School, Western Sydney University, Australia 8. David C Harris, Michael Stern Laboratory for Polycystic Kidney Disease, WIMR, USyd, Australia & Department of Renal Medicine, Westmead Hospital, WSLHD, Australia 9. Vincent Lee, Department of Renal Medicine, Westmead Hospital, WSLHD, Australia & Faculty of Medicine and Health, USyd, Australia 10. Kamal Sud, Department of Renal Medicine, Nepean Hospital, Nepean Blue Mountains Local Health District (NBMLHD), Australia & Faculty of Medicine and Health, USyd, Australia 11. Nikki Wong, Department of Renal Medicine, Nepean Hospital, NBMLHD, Australia & Faculty of Medicine and Health, USyd, Australia 12. Gopala K Rangan, Michael Stern Laboratory for Polycystic Kidney Disease, WIMR, USyd, Australia & Department of Renal Medicine, Westmead Hospital, WSLHD, Australia
Trial sponsor and contact information	Western Sydney Local Health District Human Ethics Research Committee WSLHD Research Governance Office Phone: + 61 2 8890 9007 WSLHD-researchoffice@health.nsw.gov.au
Role of the trial sponsor	The study sponsor has oversight of human ethics research committee and trial governance but no role in the design, execution, analyses, interpretation of the data or the decision to submit results
Contact for public and scientific enquires	GR [g.rangan@sydney.edu.au]
Health conditions studies	Autosomal Dominant Polycystic Kidney Disease and Hypertension

## Background

Autosomal dominant polycystic kidney disease (ADPKD) is the most common monogenic cause of kidney failure in adults and is due to pathogenic variants in either *PKD1* or *PKD2* [2]. Hypertension occurs in the third decade of life and is a crucial treatable risk factor to prevent cardiovascular morbidity and mortality in ADPKD [3–5]. Multiple pathological mechanisms drive hypertension in ADPKD; primarily renin–angiotensin–aldosterone system (RAAS) activation, endothelial dysfunction and sympathetic nervous system overactivity [3, 6].

Nitric oxide (NO) is a key mediator of vasodilation and can be produced by vascular endothelial cells via the conversion to L-arginine to L-citrulline by *nitric oxide synthase* (NOS) [6, 7]. Previous studies demonstrate that the functional loss of polycystin-1 (protein encoded by *PKD1*) leads to impaired intracellular calcium-mediated signalling within the endothelial-NOS pathway [7–11]. In a study of nine hypertensive ADPKD patients, defective endothelium-dependant relaxation was associated with an ~ eightfold reduction in NOS activity and twofold reduction in NO metabolites compared to healthy controls (*P* < 0.01) [6]. Additionally, patients with ADPKD (*n* = 27) have increased levels of asymmetric dimethylarginine (ADMA), an endogenous competitive inhibitor of NOS and independent biomarker for endothelial damage, which strongly predicts risk for future cardiovascular events [8, 12–14]. In our previous work, long-term treatment with oral sodium nitrate did not reduce kidney cyst growth in a murine genetic ortholog of ADPKD (*Pkd1*^*RC/RC*^ mice) but the effects on blood pressure (BP) could not be determined as this model does not have a hypertensive phenotype [8].

Beetroots, along with spinach, rocket and lettuce, contain the highest concentration of naturally occurring nitrate with an average of 250 mg/100 g compared with < 20 mg/100 g found in very low-concentration nitrate vegetables such as eggplants [15]. BRJ increases serum NO metabolites, promotes vasodilation and reduces BP [16–19]. The anti-hypertensive effect of BRJ is attributed to the direct conversion of dietary nitrate to NO by nitrous-converting bacteria in the oral mucosa (the entero-salivary *nitrate-nitrite-NO* pathway) [17]. Several randomised controlled trials testing the effect of beetroot juice showed a 7–12-mmHg decrease in systolic BP in individuals with hypertension, stage 3 chronic kidney disease (CKD), obesity and heart failure with preserved ejection fraction [16, 19–23]. In context, the dietary approach to stop hypertension (DASH) study showed an average reduction in systolic BP of 5.2 mmHg which, based on the 10-year Framingham risk score is associated with a 13% reduction in coronary heart disease, myocardial infarction and stroke [24, 25]. Moreover, BRJ is well accepted by study participants as it is a natural substance with no reported harmful effects and has up to 90% self-reported compliance [16, 26]. There are no expected or reported drug interactions with BRJ [16, 27–29].

Given the high burden of CVD in ADPKD patients, there is a need for safe, accessible therapies that can reduce their long-term risks [30]. BRJ may be well-suited to fulfil this need, particularly given the underlying reduction of endogenous endothelial NOS activity and NO metabolites [9, 31, 32]. To our knowledge, there have been no current or previous studies investigating the anti-hypertensive effects of BRJ in ADPKD. Thus, the aim of this study is to test the hypothesis that daily supplementation with BRJ (containing 400 mg nitrate/day) for 4 weeks will lower BP in hypertensive participants with ADPKD, using a randomised, double-blind, placebo-controlled trial design. The secondary aims are to test the effect of BRJ on home BP readings, serum and salivary NO metabolites, serum ADMA, and urinary albumin to creatinine ratio over 4 weeks.

## Methods/design

### Study objectives

BEET-PKD is a randomised, double-blind, placebo-controlled, superiority trial with 1:1 allocation of 60 participants with ADPKD and hypertension. The primary objective of this study is to evaluate the effect of 4 weeks of daily BRJ (containing 400 mg dietary nitrate/day) consumption on clinic BP in hypertensive ADPKD participants. The secondary objectives are to evaluate the effect of BRJ on daily home BP readings, nitrate metabolite levels, serum ADMA levels, and urinary albumin to creatinine ratio and monitor the safety of BRJ.

### Study sites

The study sites will be Westmead Hospital and Westmead Institute for Medical Research, Sydney, NSW, Australia. The study will be performed in accordance with the Declaration of Helsinki and strictly follow the most recent version of the study protocol that has been approved by the Human Research Ethics Committee (HREC) of the study’s sponsor, Western Sydney Local Health District (WSLHD) and meets the International Council for Harmonisation-Good Clinical Practice (ICH-GCP) guidelines. The study ethics approval number is 2020_ETH01718.

### Inclusion and exclusion criteria

A total of 60 participants who fulfil the inclusion criteria of age over 18, diagnosis of ADPKD, diagnosis of hypertension (defined as being prescribed at least one regular anti-hypertensive medication), with an estimated glomerular filtration rate (eGFR) > 30 ml/min/1.73 m^2^ (Table 1), and who do not fulfil the exclusion criteria, at the time of randomisation are eligible. The participant’s diagnosis of ADPKD will be made by the treating nephrologist based on standard criteria (family history of ADPKD and renal ultrasound). The exclusion criteria (Table 1) are based on comorbidities that could potentially confound study outcomes [33–35]. In particular, participants with severe uncontrolled diabetes (HbA1c > 10%) will be excluded due to previous studies showing that severe hyperglycemia resulted in diminished vascular responsiveness and endothelial damage [36]. Participants with well-controlled diabetes will be permitted as the incidence of type 2 diabetes in ADPKD is < 1% in the population offered in this study and it is not likely to influence the primary outcome. Participants with severe uncontrolled hypercholesterolemia and current cigarette smoking will be excluded as these states result in direct endothelial injury and suppression of nitric oxide production which will likely interfere with the action of BRJ [12, 37].

**Table 1:** Inclusion and exclusion criteria

Inclusion criteria	Exclusion criteria
• Diagnosis of Autosomal Dominant Polycystic Kidney Disease • Age > 18 years old • eGFR > 30 mL/min/1.73 m^2^ • Treatment with at least 1 anti-hypertensive	• Inability to provide informed consent • Labile, unstable, uncontrolled hypertension and/or changes in BP treatment 28 days prior to the screening visit • Non-compliance with study procedures and/or daily BP measurements during the screening period • Medical conditions or treatments that might interfere with the generation of NO metabolites or the primary endpoint (e.g. nitrate drugs, cigarette smoking; unwilling to stop using antiseptic mouthwash; severe, uncontrolled hypercholesterolaemia; pregnancy or post-partum and lactating) • Any serious or other medical conditions that might interfere with follow-up or stability of blood pressure (e.g. current active malignancies; uncontrolled diabetes mellitus with elevated HbA1c > 10%) • Dislike of taste of BRJ • Allergy to beetroot • Enrolled in other clinical trials

### Study design

**Fig. 1:**
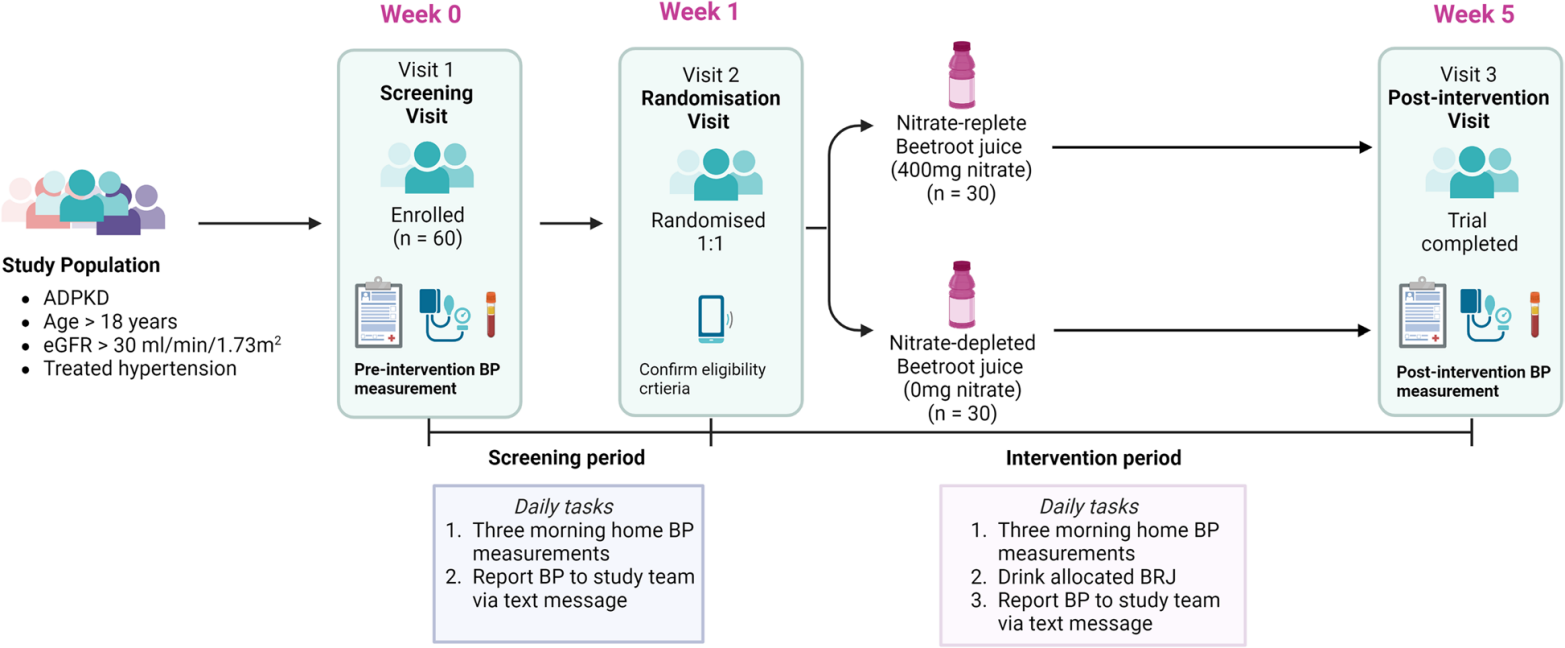
Schema of the BEET-PKD trial design. Abbreviation: *BP*, blood pressure

**Fig. 2:**
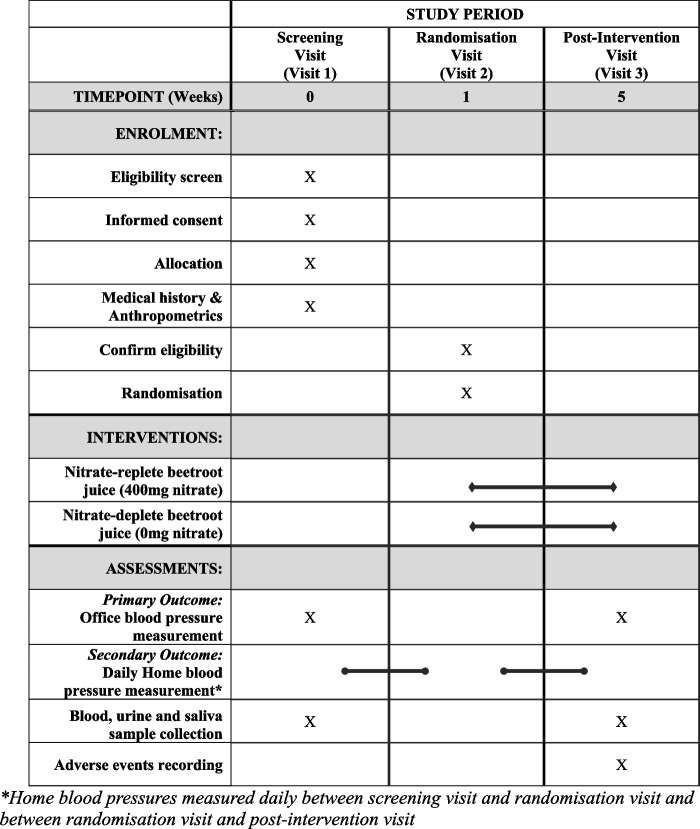
SPIRIT figure for the BEET-PKD trial

At Visit 1 participants will be screened for eligibility and instructed on correct technique for BP measurement. Pre-intervention clinic BP will be measured using a standardised protocol (Additional file 1), and blood, urine and saliva samples collected. A 1-week run-in period will be used to determine baseline home BP and adherence to daily measurements. At visit 2, BP compliance will be checked and participants will be asked to commence their randomly allocated (1:1 randomisation) BRJ daily, which is either nitrate-replete (400 mg nitrate/70 ml dose) or nitrate-depleted (0 mg nitrate/70 ml dose), for 4 weeks. Visit 2 will occur via telehealth to reduce participant burden. Over the 4-week period, participants will continue to check their BP daily and report results and any side effects by responding to the daily reminder text messages. After 4 weeks, at visit 3, participants will return for post-intervention standardised clinic BP measurement and blood, urine and saliva sample collection. Further details of the parallel assignment study design and participant’s timeline have been provided in Fig. 1 (a schema of the study design), Fig. 2 (an overview of the study schedule, interventions and procedures in a “Standard Protocol Items: Recommendations for Interventional Trials” (“SPIRIT”) figure) and Additional file 2 (a SPIRIT checklist) [1].

### Study intervention

The study intervention is a single oral daily dose of either nitrate-replete BRJ (70 mL Beet-IT Sport™; James White Drinks, UK; 400 mg nitrate/70 mL) or 70mls BRJ placebo (Nitrate-depleted Beet It shot; James White Drinks, UK) which are both identically packaged (Additional file 3). In a recent meta-analysis, 20/22 studies examining blood pressure-lowering effects of BRJ used an intervention produced commercially by James White Drinks, UK [25]. Eleven of 18 studies also used nitrate-deplete BRJ from this provider [25]. To maintain reproducibility, the current study has used the same provider. Participants will self-administer the intervention at the same time each morning within an hour of waking and prior to BP measurement. The placebo (BRJ with only nitrate content removed) was selected to exclude confounding from other vasoactive components found in BRJ such as vitamin C, magnesium, polyphenols, betaine, and flavonoids that may contribute to an antihypertensive effect [17, 38].

#### Determining dose and duration of the intervention

Previous randomised controlled trials investigating the efficacy of BRJ have used a daily dose equivalent to at least 400 mg nitrate/day [16, 17, 21, 39, 40]. In a study of treated and untreated hypertensive patients (*n* = 68), BRJ reduced BP after 1 week of daily dosing (~ 400 mg nitrate content; *p* < 0.01) and this effect was maintained for the 4-week trial duration with a mean decrease in BP of 7.7/2.4 mmHg (3.6–11.8/0.0–4.9, *p* < 0.001 and *p* = 0.050) at the end of week 4 [16]. In older (mean ages 61–62 ± 1), overweight (mean BMI 29.4 ± 1.2 and 30.5 ± 1.3) individuals with high-normal blood pressure (*n* = 24), a daily dose of BRJ (400 mg nitrate) for 3 weeks reduced daily home systolic BP (− 7.3 ± 5.9 mmHg, *p* = 0.02) during the intervention but this was not maintained 1 week after the last dose [21]. In contrast, normotensive and treated hypertensive patients (*n* = 27) treated with 1 week of daily dose of BRJ (600 mg nitrate) observed no effect [40]. Similarly, hypertensive participants with type 2 diabetes mellitus (mean HbA1c% 7.6 + 1.1%) treated with daily BRJ (450 mg nitrate) for 2 weeks showed no change in BP or endothelial function [36]. The authors reported that the lack of effect in the latter studies may be due to the relative short duration of intervention, interference of multiple pharmacological antihypertensives, low nitrate diet or chronic endothelial damage resulting in lack of vascular responsiveness to NO. Therefore for reproducibility and comparability, the current study will administer “Beet-IT Sport Shot” (70 mL; James White Drinks, UK) containing ~ 400 mg nitrate per dose for a duration of 4 weeks [39].

#### Adherence to the intervention

To improve adherence and ensure the timing of BRJ consumption and BP readings are consistent, participants will receive a scheduled daily text message through a secure web-based messaging platform (MessageMedia, Victoria, Australia) as previously described [41]. During the screening period, the message will ask participants to measure their BP and reply with their readings in the order they took the measurements. In the intervention period, the message will remind participants to take their BRJ, measure their BP and reply with the readings in the order they were measured. Adherence to the study procedures will be verified in real-time by replies to text messages.

#### Concomitant care

Participants will be advised to continue their usual medications, diet, physical activity, and other lifestyle factors as directed by their treating nephrologist. Participants must be on stable anti-hypertensive medications for 28 days prior to commencing the trial.

#### Protocol deviations

Any instances where participants discontinue or deviate from the trial protocols will be recorded as a “protocol deviation” and immediately discussed with the trial team and PI to assess for any potential SAEs. If the intervention is discontinued due to adverse events or participants’ request, participants will be encouraged to continue to record their home BP daily and attend remaining study visits and this data will be analysed as the randomised population, i.e. “intention to treat”.

### Study endpoints

#### Primary endpoints

The co-primary endpoints will be mean change in clinic systolic and diastolic BP from baseline to after 4 weeks of daily BRJ. This endpoint was selected taking into consideration the potential variability between participants’ individual BP ranges which may differ between groups and lead to biased results. This endpoint is similar to other trials of BRJ in hypertensive patients [16, 40].

#### Secondary endpoints

The secondary endpoints are mean changes in daily home blood pressures during the screening period and the 4-week intervention, and change in mean serum and salivary NO metabolites, mean serum ADMA levels, and mean urinary albumin to creatinine ratio from baseline to end of 4 weeks of daily BRJ. All adverse events will be assessed to determine the safety of the intervention (see the “Safety monitoring and reporting” section below).

### Measurement methods

#### Measurement of BP

The BP will be measured three times at clinic visits (“clinic BP”) using a validated automated oscillometric blood pressure (AOBP) device (Model: A&D UA-611, Tokyo, Japan) Standardised office blood pressure conditions will be used for all clinic readings as described in Additional file 1, adapted from the 2021 Kidney Disease Improving Global Outcomes (KDIGO) Clinical Practice Guidelines for the Management of Blood Pressure in Chronic Kidney Disease and the 2020 International Society of Hypertension Global Hypertension Practice Guidelines [42, 43]. An average of the second and third readings will be analysed for the endpoint, in accordance with the International Society of Hypertension 2020 guidelines and the 2018 European Society of Cardiology/European Society of Hypertension Guidelines [43, 44]. Home BP readings will be measured using the same AOBP device (Model: A&D UA-611, Tokyo, Japan) which will be provided to the participants for the duration of the trial. At the first visit, participants will be trained on how to correctly measure their blood pressure following the Heart Foundation of Australia’s self-measurement instructions and a detailed instruction card on the correct technique which will be attached to the device [45]. As with the clinic BP, home BP will be measured three times and an average of the second and third reading used for data analysis.

#### Measurement of urinary albumin:creatinine ratio, NO metabolites and ADMA

Serum samples to measure NO metabolites and ADMA levels, and saliva samples to measure NO metabolites will be collected at visit 1 and the final visit (visit 3) on the last day of the intervention. Urine samples for urinary albumin to creatinine ratio will be collected at visit 1 (or if participants have recently completed a measurement at an accredited lab in the 6 months prior to the visit, this measurement may be used) and at visit 3 on the last day of the intervention. See Additional file 4 for details on the collection, storage and methods of measurement of biological samples.

### Determination of sample size

In a previous study of daily BRJ (400 mg nitrate) supplementation in hypertensive patients (*n* = 68) for 4 weeks, systolic BP was significantly reduced by 7.7 mmHg (3.6–11–8 mmHg, *p* < 0.001) at the end of the intervention [16]. The average baseline BP of the two groups were 148 mmHg and 149 mmHg, with standard deviations of the two populations being 10 and 11. We expected to have a similar hypertensive population and a test for differences in two independent means was implemented in Stata SE Version 14.2 (StataCorp, TX, U.S.A) to calculate sample size. To detect this difference in systolic BP with *α* = 0.05 and power of 0.80, 28 participants are required in each treatment group. To account for drop-out, the sample size was increased to 30 participants per treatment group (total *n* = 60). Participants will be referred from the Western Renal Service which provides a catchment of 1.2 million people and 400 potential participants with ADPKD, and therefore a single-centre study with multiple referral centres was considered adequate for this study.

### Recruitment

Participants will be recruited from the Western Renal Service (Westmead, Blacktown and Nepean hospitals) which services a catchment area of 1.2 million people and 400 potential participants with ADPKD. If required, other local centres such as Concord, Royal North Shore and Liverpool Hospitals. Multiple strategies will be used to facilitate recruitment. Potential participants will be identified from the Principal Investigator’s patient database and databases of previous clinical trial participants who have opted-in to be contacted about future trials. Potential participants will also be identified from treating nephrologists, either through direct referral to the study team or review of clinic letters and local databases. Participants will also be recruited passively by advertising through the PKD Australia’s website and newsletter.

Study staff (study doctors or senior researchers) will obtain written informed consent from all participants prior to commencing the trial. All participants will receive the consent form and information about the trial prior to visit 1 and are able to contact the study team with questions. During visit 1, study staff will go through the information and consent forms and have an informed discussion with each participant. Specifically, participants will be informed of the expected adverse effects of beeturia and beet-coloured faeces [17]. Participants will also be informed of the potential for gastrointestinal side effects and asked to report any symptoms to the trial team immediately.

### Randomisation and blinding

Participants will be randomised using a simple randomisation program created by the study biostatistician, which uses a random number generator (version 3.6.2, R Core Team). This program creates a list that allocates treatment or placebo (1:1) to 60 unique randomisation IDs and will be generated by the study biostatistician. No stratification factors or blocking will be used. When a participant is randomised, they will be assigned a randomisation ID. Prior to commencement of the trial, a research staff member who is not involved with any other study procedures will receive the randomisation IDs and label the relevant BRJ with the study ID as allocated by the list. The study team will provide the participant with their labelled BRJ at Visit 1. The remaining members of the study team and the participants will remain blinded to the treatment allocation until data lock and statistical analysis have been completed. Emergency unblinding procedures will only occur if the safety of trial participants is at risk or should any evidence of harms arise, as per the process described below (in the “Safety monitoring and reporting” section and Additional file 6).

### Statistical analysis plan

#### Primary endpoint analysis

The co-primary endpoints are the change in the mean of second and third systolic and diastolic BP measurements taken at Visit 1 (pre-intervention) and at Visit 3 (at the end of week 4 of the intervention). Initially, the sample mean and standard deviation will be reported at baseline and follow-up. Moreover, their mean difference and standard deviation will be reported. Secondly, the main analysis will be conducted using a Gaussian linear mixed effects (LME) model with a first-order autoregressive correlation structure (AR(1)). The repeated measures are the BP results at Visit 1 and Visit 3. An interaction parameter between the visit and arm of the study will be the determinant of a significant difference in BP change between the two arms. The model will be adjusted for the following prognostic factors: age, eGFR and baseline serum nitrate/nitrite levels. The sensitivity analysis will only be adjusted for age and eGFR. Should any variables differ significantly between the two arms, they too will be adjusted for to eliminate the possibility of a confounding effect. No subgroup analyses will be undertaken. All data analysis will be completed in R version 3.6.2 (R Core Team). All (potential) confounding variables will be summarised using *t*-tests or rank sum tests for continuous variables and chi-squared tests or Fisher’s exact tests for categorical variables. Means (standard deviations) and counts (column percentages) as well as relevant p-values will be reported. Hypotheses will be conducted with a two-sided alternative and p-values less than 0.05 will be considered statistically significant*.*

#### Secondary endpoint analysis

The secondary endpoint of home BP readings is a set of (at most) 28 daily systolic and diastolic BP measurements averaged from daily home readings. Again, a Gaussian LME model with an AR(1) correlation structure will be utilised. The repeated measures are the BP results at each available home reading. An interaction parameter between the day and arm of the study will be the determinant of a significant difference in the BP change between the two arms. The model will be adjusted for the prognostic variables of age, eGFR and baseline nitrate/nitrite levels as described above. The secondary endpoints of change in serum and salivary NO metabolites, serum ADMA and albumin to creatinine ratio will have their differences summarised with sample means, standard deviations, sample mean differences, and sample standard deviations of differences. They too will undergo assessment with a Gaussian LME model as above.

#### Missing values

Missing values will not be imputed. An advantage of LME models is that they can automatically tolerate missing values by adjusting the respective covariance estimates. Moreover, missing data for the primary outcome is expected to be minimal as this BP data will be collected at the study visits by the investigators.

### Data collection and storage

All participants will receive a unique study ID for data collection and analysis. Visit data will be collected on paper-based forms which will be stored in a secure location and will also be scanned to the sponsor’s secure server. Visit forms, screening pathology, home BP readings, protocol deviations and adverse outcome reports will be de-identified with the participant study ID and stored on a database on the same secure server. Any identifying information will be stored separately and securely with controlled access.

### Data monitoring

BRJ is a food supplement that is readily available for consumption from commercial food outlets. Reported side effects are non-harmful pigmentation of faeces and urine (beeturia) [17]. Previous clinical trials have not reported any serious adverse effects (SAEs) and thus they are anticipated to be rare in this trial and no interim analyses will be undertaken [17, 25]. Due to the nature of the intervention and the size of the trial, the data monitoring will be undertaken by the trial investigators (who have no competing interests to declare) and there are no interim analyses or audits planned. All data will be stored securely in the Western Sydney Local Health District and will be available for audit as requested by the Human Research Ethics Committee. The funding body has no role in the data monitoring.

### Safety monitoring and reporting

The trial staff will monitor for adverse effects or other trial issues at each study visit systematically by asking participants if they have experienced any symptoms, and these will be reviewed in at least fortnightly meetings between the principal investigator (PI) and trial staff. All SAEs will be reported immediately to the PI and the WSLHD HREC, investigated in detail and documented in accordance with the ICH-GCP guidelines. All adverse events will be graded by consensus amongst investigators and reported using the “BEET-PKD Adverse Event Reporting Guide” on an adverse event form (guide and example form provided in Additional file 5). The decision to stop or change the intervention is made by the PI and trial team and is based on the severity of the individual participant’s symptoms and, in the setting of mild non-harmful adverse events, the participant’s wishes. In the setting where the intervention is changed, it is reported as a protocol deviation. If there is a SAE, unblinding will occur according to the flowchart in Additional file 6 (adapted from the University of Leicester, UK) and the unblinded research staff will reveal the relevant participant’s allocation to the PI. The decision to terminate the trial will be taken by the PI and WSLHD HREC and will be based on the review of SAEs. Any reported adverse events related or not related to the intervention will be reported in the publication of trial results.

### Post-trial care

As all participants currently receive specialist care within public hospitals and given the nature of BRJ, with its limited and mild side effect profile reported in previous clinical trials (beet-coloured pigmentation of urine and faeces), the investigators do not anticipate a need for additional special provisions for post-trial care [17].

### Trial registration

Ethics approval for this study was obtained in May 2021. A submission to the clinical trials registry (ClinicalTrials.gov) was uploaded on 5th April 2022, pre-dating the recruitment of the first participant on 5th May 2022, but was not finalised pending HREC approval of a minor amendment. The trial registration was finalised on 27th May 2022 (registration no: NCT05401409).

### Protocol amendments

The study was approved by the Western Sydney Local Health District Human Research Ethics Committee (Approval no. 2020_ETH01718). The investigators will seek approval of the WSLHD HREC prior to the implementation of any changes to the study protocol and notify the health authorities in accordance with local regulations if required. Trial participants, trial registries, journals, and study investigators will be notified as appropriate. Minor changes and clarifications of the protocol that have no impact on the conduct of the study will be documented in a memorandum.

### Dissemination of results

The results of the study will be disseminated at national and international scientific meetings and submitted for publication in peer-reviewed journals. Participants will be notified when results of the study are available to the public.

## Discussion

There is currently no cure for ADPKD and the search for therapies that will slow disease progression is a major priority for PKD patients, community groups and healthcare providers [31, 32]. In particular, there is interest in developing dietary and lifestyle interventions for ADPKD, and this was highlighted in a recent patient priorities survey, where it was ranked 7 out 17 of major research priorities [32].

BRJ is an attractive novel therapy in the treatment of hypertension as it is all-natural, accessible and has no adverse side effects in human studies to date. There are no expected or reported drug interactions with beetroot juice [28, 29, 46]. There has only been one RCT describing the effects of BRJ in CKD and no studies on an ADPKD cohort [19]. As described earlier, there is an inherent reduction in NO and endothelial NOS activity in ADPKD, which could potentially be corrected by dietary nitrate supplementation via the entero-salivary *NO-nitrite-nitrate* pathway [6, 8]. Previous studies have shown that supplementation with BRJ increases NO metabolites in healthy volunteers and chronic diseases including essential hypertension, diabetes, heart failure, and COPD [16, 20, 36, 47]. Of particular interest, BRJ increases NO metabolites in hypercholesterolemia, which is associated with decreased NO endothelial production, via this alternate entero-salivary pathway [22]. This pathway appears to be attenuated in other conditions such as active smoking, likely due to direct inhibitory effects [37]. This trial aims to investigate if NO metabolites can be increased using BRJ in ADPKD, given its intrinsic decreased NO state, and furthermore, if that will result in a reduction in BP.

In conclusion, this study will be the first RCT investigating the efficacy of BRJ in reducing BP in hypertensive participants with ADPKD. If the results show a significant decrease in BP, a follow-up study would be warranted to test the efficacy of BRJ over a longer time course and evaluate the potential impacts on chronic cardiovascular outcomes in ADPKD. Moreover, if the results show that NO can be increased with BRJ supplementation, other sources of dietary nitrate (e.g. spinach, rocket, lettuce) could also be explored for BP-lowering effects. Additionally, this study will contribute to the existing evidence on the impacts of dietary interventions in managing chronic kidney diseases, an area of keen interest from the patient community.

## Trial status

The current study protocol (version 7) was accepted on 27th June 2022 by the Western Sydney Local Health District Human Research Ethics Committee. Trial recruitment commenced on 5th May 2022 and was completed on 24th March 2023. The trial was registered on 27th May 2022 with ClinicalTrials.gov (NCT05401409), URL: https://clinicaltrials.gov/ct2/show/NCT05401409.

## Supplementary Information

**Additional file 1.** Standardised clinic measurement of blood pressure in the BEET-PKD clinical trial. Description: Table describing the standardisation of blood pressure measurement in the BEET-PKD trial.

**Additional file 2.** SPIRIT checklist. Description: Checklist for key study protocol elements in this manuscript

**Additional file 3.** Photo of nitrate-replete and nitrate-deplete beetroot juice bottles. Description: Photo of nitrate-replete and nitrate-deplete beetroot juice bottles.

**Additional file 4.** Storage and analysis of biological samples in the BEET-PKD clinical trial. Description: Document describing the storage and analysis of biological samples in the BEET-PKD clinical trial.

**Additional file 5.** BEET-PKD Adverse Event Reporting Guide and Example form. Description: Adverse event reporting guide used by Investigators and Blank adverse event reporting form

**Additional file 6.** Flowchart to guide unblinding in the BEET-PKD study. Description: Flowchart to guide unblinding in the BEET-PKD study (adapted from the University of Leicester, United Kingdom).

## Data Availability

The results of the datasets have not been reported in this manuscript. Details of data accessibility (including the public access to the full protocol, model consent form, participant datasets and statistical code) will be included in the publication containing the final results of the study.
