# Supplementary material for: Efficacy of beetroot juice on reducing blood pressure in hypertensive adults with autosomal dominant polycystic kidney disease (BEET-PKD): study protocol for a double-blind, randomised, placebo-controlled trial
Source: Trials. 2023 Jul 29;24:482. doi: 10.1186/s13063-023-07519-2 (PMC10386227; doi:10.1186/s13063-023-07519-2)
Supplement: Supplementary file 1 — Additional file 1. Standardised clinic measurement of blood pressure in the BEET-PKD clinical trial. Description: Table describing the standardisation of blood pressure measurement in the BEET-PKD trial. [file 13063_2023_7519_MOESM1_ESM.pdf]

Additional File 1: Standardised clinic measurement of blood pressure in the BEET-PKD clinical trial.

Environment	• Quiet clinical space<br>• The investigator will leave the room once the participant is appropriately prepared and comfortable with using the BP machine
Participant preparation and equipment	• Participant is sitting in a chair with feet on the floor and back supported<br>• Relaxed, in the sitting position for at least 5 minutes prior to measurement<br>• Not speaking during the rest period or during the measurement<br>• Arm exposed so that cuff is fitted over bare skin<br>• Arm is resting on a table roughly at heart level<br>• Validated AOBP device (Model: A&D UA-611, Tokyo, Japan) used<br>• Correct cuff size used so that 80% of the arm is covered by the cuff
Measurement	• The same arm is used for all clinic and home measurements (arm choice is based on which arm the participant is most dextrous and comfortable with to ensure correct technique with unobserved home BP measurements)<br>• Measure BP three times with 1 minute interval between readings
Data analysis	• An average of the second and third readings will be taken for outcome analysis

BP, blood pressure; AOBP, automated oscillometric blood pressure. Adapted from Kidney Disease: Improving Global Outcomes (KDIGO) Blood Pressure Work Group. KDIGO 2021 Clinical Practice Guideline for the Management of Blood Pressure in Chronic Kidney Disease. Kidney Int. 2021;99(3S):S1–S87.
