# Supplementary figures and images for: Efficacy of beetroot juice on reducing blood pressure in hypertensive adults with autosomal dominant polycystic kidney disease (BEET-PKD): study protocol for a double-blind, randomised, placebo-controlled trial
Source: Trials. 2023 Jul 29;24:482. doi: 10.1186/s13063-023-07519-2 (PMC10386227; doi:10.1186/s13063-023-07519-2)

**Additional File 3: Photo of nitrate-replete and nitrate-deplete beetroot juice bottles.**

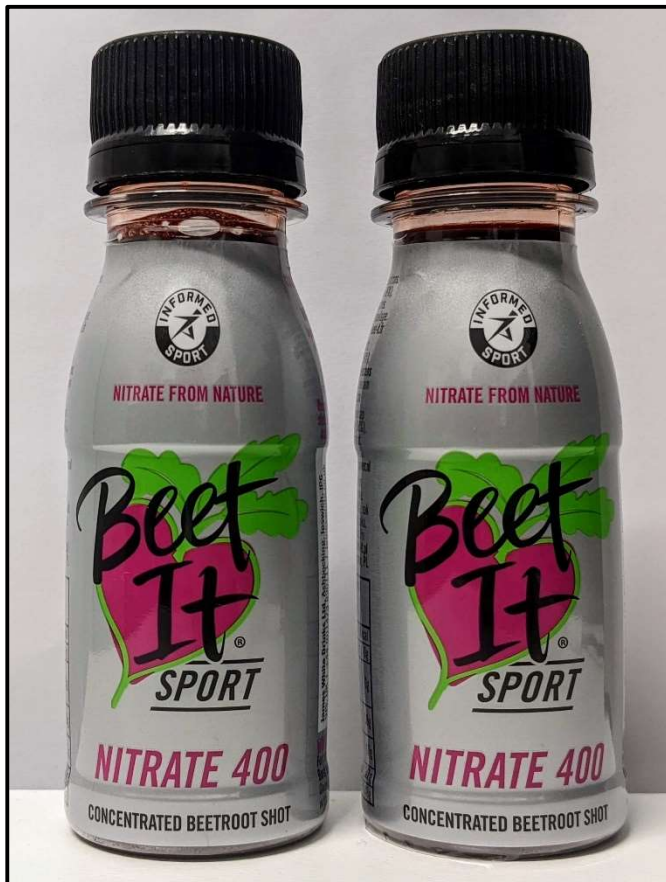

Supplement: Supplementary file 3 — Additional file 3. Photo of nitrate-replete and nitrate-deplete beetroot juice bottles. Description: Photo of nitrate-replete and nitrate-deplete beetroot juice bottles. [file 13063_2023_7519_MOESM3_ESM.pdf]
