# Supplementary material for: Efficacy of beetroot juice on reducing blood pressure in hypertensive adults with autosomal dominant polycystic kidney disease (BEET-PKD): study protocol for a double-blind, randomised, placebo-controlled trial
Source: Trials. 2023 Jul 29;24:482. doi: 10.1186/s13063-023-07519-2 (PMC10386227; doi:10.1186/s13063-023-07519-2)
Supplement: Supplementary file 4 — Additional file 4. Storage and analysis of biological samples in the BEET-PKD clinical trial. Description: Document describing the storage and analysis of biological samples in the BEET-PKD clinical trial. [file 13063_2023_7519_MOESM4_ESM.docx]

**Additional File 4:** **Storage and analysis of biological samples in the BEET-PKD clinical trial**

*Preparation and storage of samples*

Blood,urine and saliva samples will be collected at Visit 1 and 3 for urinary albumin to creatinine ratio (ACR), NO metabolites and ADMA levels. Blood will be collected in a gel separator tube and allowed to clot. The tube will then be centrifuged at 1000xg for 15 minutes after which the serum will be aliquoted and stored at -30⁰C. Urine will be transported to the lab on ice. 25mls of the urine sample will be sent for formal laboratory measurement of urinary ACR (Institute of Clinical Pathology and Medical Research (ICPMR), Westmead Hospital). The remaining urine sample will be centrifuged at 1000xg for 5 minutes. 12.5mls of urine will be mixed with 50mg sodium hydroxide (as a preservative), the remaining sample will be aliquoted and all samples will be stored at -30⁰C. Saliva samples will also be collected for future post-hoc analysis of oral microbiome.

*Analysis of serum and salivary nitrate/nitrite by* *enzyme-linked immunosorbent assay (ELISA)*

Nitrate/nitrite analysis of serum and saliva will be performed using commercially available colorimetric Nitric Oxide Assay kits, based on the Griess assay, according to the manufacturer’s instructions. Briefly, nitrate is reduced to nitrite by the addition of nitrate reductase to the plasma and saliva samples, followed by the addition of Griess reagent to form a deep purple azochromophore for measurement of nitrate/nitrite using a microplate reader (OD 540 nm).(1) Saliva samples will be deproteinised prior to the initial reduction step, as recommended by the manufacturer.

*Analysis of serum asymmetric dimethylarginine (ADMA) by ELISA*

Serum ADMA analysis will be performed using a commercially available quantitative sandwich ELISA kit, according to the manufacturer’s instructions. Briefly, the sample is added to a microplate coated with immobilised anti-ADMA antibodies to bind to ADMA present in the sample. Unbound substances will then be removed, followed by the addition of biotin conjugated ADMA specific antibodies. A substrate solution is then added and absorbance at 450 nm will be measured using a microplate reader to determine the amount of ADMA in the samples.

**References**

1. Tsikas D. Analysis of nitrite and nitrate in biological fluids by assays based on the Griess reaction: appraisal of the Griess reaction in the L-arginine/nitric oxide area of research. J Chromatogr B Analyt Technol Biomed Life Sci. 2007;851(1-2):51-70.
