# Supplementary material for: Efficacy of beetroot juice on reducing blood pressure in hypertensive adults with autosomal dominant polycystic kidney disease (BEET-PKD): study protocol for a double-blind, randomised, placebo-controlled trial
Source: Trials. 2023 Jul 29;24:482. doi: 10.1186/s13063-023-07519-2 (PMC10386227; doi:10.1186/s13063-023-07519-2)
Supplement: Supplementary file 5 — Additional file 5. BEET-PKD Adverse Event Reporting Guide and Example form. Description: Adverse event reporting guide used by Investigators and Blank adverse event reporting form [file 13063_2023_7519_MOESM5_ESM.pdf]

1. An AE is any untoward medical occurrence, unfavourable and unintended sign, symptoms or disease in the participant temporarily associated with the clinical study whether or not considered related to the procedures. AEs will be documented from the time of randomization until the final visit. Changes to health between the time of consent and the time of randomization will be recorded as Medical History.
    a. Procedures are defined as intervention and or study requirements e.g. blood tests, MRI.

2. Any AE that result in the following outcomes is considered as serious:
    a. Death
    b. Life-threatening
    c. Requires in-patient hospitalization OR significant testing or treatment upon presentation to ED OR if ambulance is called by participant
        i. Significant testing or treatment= beyond triage, beyond pain relief or prescription given (e.g. given IV antibiotics or pain relief or fluids OR CT scan/other imaging)
        ii. Please note, the AE or AEs that prompted presentation to the hospital should only be labelled serious. E.g. if participant has cough and then develops macrohaematuria and fever, presents to ED because of macrohaematuria and fever, then only these should be marked as serious despite concomitant cough.
    d. Significant disability or incapacity
    e. Congenital abnormality or birth defect
    f. Medically significant e.g. ESRF, doubling of serum creatinine, significant drop in kidney function

3. Intensity:
    a. Mild – easily tolerated, causing minimal discomfort and not interfering with everyday activities.
        i. Asymptomatic/incidental finding/symptoms not affecting usual lifestyle or able to take (For example) paracetamol and carry on with usual day/activities
    b. Moderate – sufficiently discomforting to interfere with normal activities.
        i. Symptoms prompt time off work(still able to complete ADLs)/GP/Allied health visit and/or requires a prescription treatment or requires day surgery
    c. Severe – prevents normal everyday activities.
        i. Symptoms prompt time off work (bedridden) (with/without GP visit) or presentation to hospital or requires surgery/operation as an outpatient

    NB: Where classifying AEs where all necessary information was not collected at time of reporting and is unable to be reported or not remembered by participant, the AE should be classified to the best of ability.

4. Causality:
    a. Not related – no causal relationship between the study procedures/intervention and the AE.
    b. Unlikely- temporally associated with study procedures/interventions but are not likely to have any reasonable association with the AE.
    c. Possible – temporally associated with the study procedures/intervention but could have been caused by patient's clinical state or other modes of therapy administered.
    d. Definite – temporally associated with study procedures/intervention, abates upon discontinuation of procedure/ intervention and reappears when reintroduced (e.g. Beeturia)

Additional Notes

1. What constitutes an AE?
    a. Any PKD related checklist finding – new condition and worsening are separate AEs
    b. Any change in normal or expected health of participant (From participant perspective)
    c. Any pain
    d. Any event in which procedures are completed, investigations done or treatment provided
    e. Any signs or symptoms (in some cases even if self-reported, e.g. refer to Oedema)
    f. Any official diagnoses by participant's doctor or written in a discharge summary
    g. Any 'flare-up' or worsening of symptoms (increase in frequency or intensity) of a chronic condition noted in medical history
        i. Marked as ongoing if no specific/noticeable ceasing of symptoms
    h. Any new conditions detected or diagnosed after randomization even though it may have been present prior to the start of the study (e.g. liver cysts identified at Month 18 MRI where participant had not noted them in screening, and were not reported in screening MRI)
    i. Any conditions or events noted in a doctors letter that the participant has failed to/not remembered to disclose.
    j. Where a participant has one AE that is then diagnosed (e.g. flank pain > pyelonephritis, bursitis > arthritis), then the AE is labelled the former symptom or diagnosis up until the diagnosis or confirmed diagnosis. The end date of the former (e.g. flank pain) is the date of diagnosis of the latter (pyelonephritis). There are many reasons for this including: diseases/illnesses have varied signs and symptoms and merely labelling as the disease state does not capture this; even if a symptom is associated with a diagnosis, it might not be related to that and the role of the study/intervention should still be considered – having only a diagnostic AE or disease term does not allow for this scrutiny; promotes integrity by only recording diagnoses or associations between symptoms/signs and condition which are official and complete by the participants

medical team/the investigator and study team does not assume. Please also note that where symptom led to medically significant event (e.g. hospitalization) and then later clinical impression /diagnosis made, both are considered AEs, both are considered serious.

2. Start and End Date
    a. Where a participant cannot remember an AE date, a proxy date will be used that as closely resembles as possible when the event occurred. For example (1) "It happened Mid May 2019" = 15/05/2019 or (2) "It happened start of May 2019" = 01/05/2019.
    b. This extends to end dates where a participant cannot remember exactly how long symptoms occurred for or when they resolved. For example (1) "It happened Mid May and lasted a few days" = 15/05/2019-18/05/2019 or (2) "I had a cold at the end of May and it lasted a couple of days" = 31/05/2019-2/05-2019.
    c. Where participants cannot give a rough indication of how long symptoms occurred for or when they resolved OR when participants are still experiencing the AE at the time of current visit where it is reported and if the AE is not reported at the consequent study visit, the date of the consequent study visit can be entered as end date. For example (1) Pt. reported "I have a sinus infection at the moment" at Visit 3 (01/05/19). Pt. did not report sinus infection at Visit 4 (01/06/19), but cannot remember when symptoms ceased, end date of sinus infection is (01/06/19).
        i. This is to ensure we are capturing the longest period for which symptoms could have been present
3. Is it a PKD related event? (Please refer to table below for specific AEs and examples)
    a. Any findings from the PKD checklist are PKD related events.
    b. Pain (abdominal, flank and back) that have no other reasonable explanation can be considered PKD related.
    c. Often TBC based on Investigator interpretation / refer to AE classification table.
    d. If an AE occurs as a result of Tolvaptan e.g. polyuria, then it is *not* a PKD related event.
4. Treatment or diagnostic procedure
    a. All investigations, procedures or treatments should be entered on concomitant medication form.
    b. If treatments are unknown but the AE could not be resolved otherwise according to medical judgement, 'unspecified treatment' or 'unspecified medical treatment' or 'unspecified hospital treatment' can be entered as a proxy.
    c. If participant sees a specialist/doctor and cannot remember investigations or procedures done, please enter 'Specialist review' for example.
    d. If participant sees an allied health practitioner, this should be recorded. For example as 'physio treatment'.
    e. All OTC supplements should be captured. If they are not taken for medical need or taken without symptoms, indication is "wellbeing"

BEET-PKD Study Adverse Event Form

Screen ID S______ Randomization ID R______

**Event Term**	
**Narrative**	
**Start Date**	
**Is this a PKD disease related event?**	
**Is this a serious event (SAE)?**	
**Date when the event became serious:**	
**Seriousness criteria**	☐ Result in death<br>☐ Life-threatening<br>☐ Requires or prolongs hospitalisation<br>☐ Results in disability or incapacity<br>☐ Result in congenital anomaly or birth defect<br>☐ Medically significant:
**Intensity**	☐ Mild<br>☐ Moderate<br>☐ Severe
**Action taken with study treatment**	☐ Unchanged/no action<br>☐ Withdrawal of treatment<br>☐ Dose increased<br>☐ Dose decreased
**Causality**	☐ Not related<br>☐ Unlikely<br>☐ Possible<br>☐ Definite
**Any treatment or diagnostic procedure given?**	
**Outcome**	☐ Fatal<br>☐ Ongoing<br>☐ Permanent residual effect<br>☐ Resolved
**Resolved date**	
