# Supplementary material for: Efficacy of beetroot juice on reducing blood pressure in hypertensive adults with autosomal dominant polycystic kidney disease (BEET-PKD): study protocol for a double-blind, randomised, placebo-controlled trial
Source: Trials. 2023 Jul 29;24:482. doi: 10.1186/s13063-023-07519-2 (PMC10386227; doi:10.1186/s13063-023-07519-2)
Supplement: Supplementary file 6 — Additional file 6. Flowchart to guide unblinding in the BEET-PKD study. Description: Flowchart to guide unblinding in the BEET-PKD study (adapted from the University of Leicester, United Kingdom). [file 13063_2023_7519_MOESM6_ESM.pdf]

Additional File 6: Flowchart to guide unblinding in the BEET-PKD study (adapted from the University of Leicester, United Kingdom)

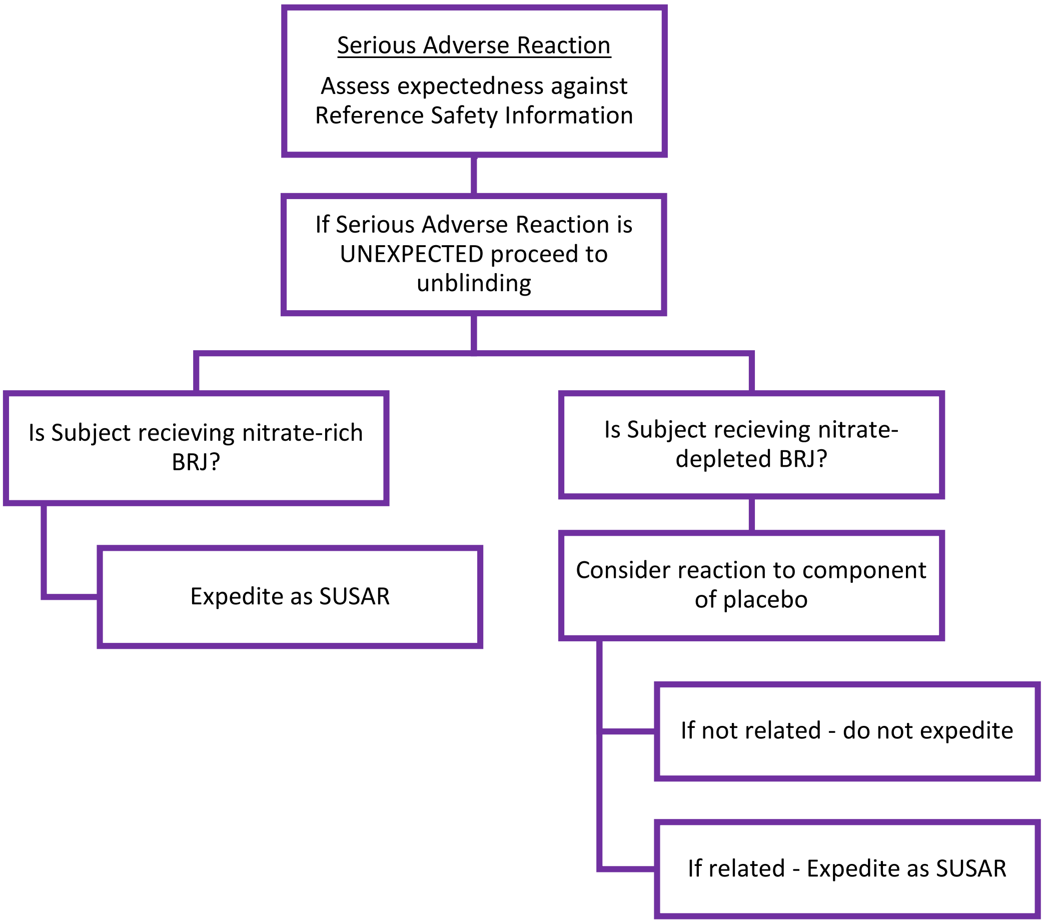

Abbreviations: SUSAR; suspected unexpected serious adverse event
